# The Importance of Good Measurement: Development and Validation of a Measure of Disordered Eating Among Adults with Chronic Pain

**DOI:** 10.1080/24740527.2023.2284815

**Published:** 2024-02-26

**Authors:** E. Lamoureux, M. G. Pagé

**Affiliations:** Research Center, Centre de Recherche du Centre Hospitalier de l’Université de Montréal, Montreal, Quebec, Canada; Department of Psychology, Faculty of Arts and Sciences, Université de Montréal, Montreal, Quebec, Canada; Department of Anesthesiology and Pain Medicine, Université de Montréal, Montreal, Quebec, Canada

Many individuals living with chronic pain seek treatment and engage in various pain-related coping strategies to minimize its impact.^1^ Pain-related coping strategies comprise active and passive cognitive, behavioral, and emotional responses to pain, and can be classified as either adaptative or maladaptive as a function of their effect on overall functioning.^2^ Growing literature now supports the association between maladaptive pain coping strategies such as pain catastrophizing, pain avoidance and pain-induced comfort eating, and poorer pain outcomes as illustrated by greater pain intensity^3,4^ and psychological distress.^5–7^ New scientific knowledge on the association between some of these maladaptive coping strategies and various dimensions of chronic pain is facilitated by the availability of psychometrically sound self-reported measures to document these cognitive, emotional and behavioral responses. As such, the literature associated with some of these constructs, such as pain catastrophizing, is much more developed than for others, such as pain-related emotional eating. To our knowledge, only one measure of pain-related emotional eating exists,^8^ and Burton and colleagues^9^ propose the first evaluation of its psychometric properties.

Indeed, in their recent article “Development and Validation of the Pain-induced Comfort Eating Scale in a Chronic Pain Sample,” Burton and colleagues sought to establish the reliability and validity of the Pain-induced Comfort Eating Scale (PICES).^9^ Pain-induced comfort eating is defined as the tendency to overeat pleasurable foods to cope with the discomfort associated with pain.^8^ Interestingly, many studies have shown that emotional eating is a fairly common pain coping strategy among individuals with chronic pain.^3,4,10^ One possible explanation for these findings is that foods that have a high sugar and fat content provide an analgesic effect and promote pain tolerance.^11–13^ Having a reliable and valid measure of pain-induced comfort eating could facilitate the identification of early signs and symptoms of disordered eating and of maladaptive coping strategies based on high food intake to deal with the physical and psychological discomfort associated with pain flare ups among patients with chronic pain. And Burton’s and colleagues’ study offers preliminary data on the psychometric performance of the PICES. The authors acknowledge that this scale validation was performed using secondary analyses of an available dataset that was not designed or built for the purpose of scale validation. One must thus wonder whether using the scale in its current state knowing the current study’s limitations, would help us further our knowledge on the role of pain-induced comfort eating in chronic pain experiences.

Best practices for scale evaluation include tests of dimensionality, reliability, and validity,^14^ none of which were fully explored in this study. Tests of dimensionality refers to testing the hypothesized factor structure extracted from a previous model, ideally in a new sample, and longitudinally.^15^ It is typically done through confirmatory factor analysis, bifactor modeling, or measurement invariance. In Burton’s study, they instead conducted an exploratory factor analysis that is typically part of a scale’s development rather than validation. Tests of reliability were also incompletely performed, since only Cronbach’s alpha was presented, with no indication of the scale’s test-retest reliability. And finally, the last step of a scale’s evaluation is the test of its validity, which includes criterion validity and construct (convergent and divergent) validity. The PICES’ convergent validity was assessed by correlating the scale’s total score with measures of disordered eating, emotional eating, average pain intensity, pain catastrophizing, and psychological distress. Now, it is important to remember that this scale evaluation was conducted using an available dataset that did not allow authors to select new measures that might have provided optimal information about the scale’s construct validity. But one must wonder whether using this convenient approach is good enough. The first two scales (disordered eating and emotional eating) are really closer to criterion validity (how the PICES compared to a gold standard) than construct validity. Also of note is the theoretical basis from which moderate correlations with the Depression Anxiety and Stress Scale-21 (DASS-21; a reliable and validated measure of depression, anxiety and stress) was used to conclude that the scale has good convergent validity, as it does not assess maladaptive behavioral pain coping strategies. We question the extent to which this measure showcases convergent validity with pain-induced comfort eating.

Importantly, the authors argue that the PICES might be helpful to understand maladaptive ways individuals cope with pain flares; yet they assessed this using an average measure of pain intensity. While O’Loughlin & Newton-John^8^ operationalized pain flares as “an exacerbation of [the patient’s] usual pain levels” in the PICES instructions, a study by Setchell and colleagues^16^ investigating the semantic concept of pain flare among patients with low back pain found that over 50% of the participants did not consider a flare to be synonymous with a pain increase or were unsure about it. Given that average pain intensity was not associated with the PICES, it is imperative that future studies seek to confirm whether the PICES flare item correlates with measures of pain flare frequency, intensity, and interference. We also note that analyses of convergent validity can only provide partial information about a scale’s validity, and that a good research practice is to examine it in conjunction with discriminant validity, as both are complementary indicators of construct validity.

As noted by the authors, there are also important limitations regarding the generalizability of their findings given how their sample was composed. Over 95% of the recruited participants identified as female, over 80% also identified as Caucasian and over 55% had obtained a bachelor’s degree or higher. While chronic pain tends to be significantly higher in women, research indicates higher prevalence of pain among people with lower education levels and socioeconomic status in Australia and across the world.^17^ Further studies have also shown substantial ethnic and cultural differences in the prevalence, treatment and outcomes of chronic pain conditions,^18–20^ showcasing the importance of considering the effects of race and ethnicity on the pain experience in health care settings and research.^21^

Overall, O’Loughlin & Newton-John^8^ aimed to create a measure of pain-related emotional eating behavior which is currently lacking in the field of pain science and which would shed light on the relationship between chronic pain and disordered eating behaviors. We argue however, that the initial validation by Burton and colleagues^9^ doesn’t provide enough evidence to support the validity and reliability of the PICES at this stage. While such novel assessment tool allows the research community to better understand the wide range of pain impacts and is a very important endeavor, we need to ensure that they are carefully developed and validated, otherwise we don’t really know what the scale measures. Extensive methodology and guidelines have been published to inform scale development, yet an increasing number of scales are being developed based on research studies that have significant methodological imitations.^22^ Yes, we need to start somewhere when we develop a scale and not all aspects of scale development and validation can be performed within a single study. There is a risk however, in using a scale that has yet to show adequate psychometric properties.
